# Rates of family history of autism and ADHD varies with recruitment approach and socio‐economic status

**DOI:** 10.1111/bjdp.12469

**Published:** 2023-11-16

**Authors:** Tessel Bazelmans, Gaia Scerif, Karla Holmboe, Nayeli Gonzalez‐Gomez, Alexandra Hendry

**Affiliations:** ^1^ Institute of Psychiatry, Psychology and Neuroscience King's College London London UK; ^2^ Department of Experimental Psychology University of Oxford Oxford UK; ^3^ School of Psychological Science University of Bristol Bristol UK; ^4^ Department of Psychology, Health and Professional Development Oxford Brookes University Oxford UK

**Keywords:** family history, neurodevelopmental conditions, prevalence, socio‐economic status

## Abstract

Family history (FH) of autism and ADHD is not often considered during the recruitment process of developmental studies, despite high recurrence rates. We looked at the rate of autism or ADHD amongst family members of young children (9 to 46 months) in three UK‐based samples (*N* = 1055) recruited using different methods. The rate of FH‐autism or FH‐ADHD was 3%–9% for diagnosed cases. The rate was highest in the sample recruited through an online participant pool, which also consisted of the most socio‐economically diverse families. Lower parental education and family income were associated with higher rates of FH‐ADHD and lower parental education with increased FH‐autism. Thus, recruitment strategies have a meaningful impact on neurodiversity and the conclusions and generalizations that can be drawn. Specifically, recruitment using crowdsourcing websites could create a sample that is more representative of the wider population, compared to those recruited through university‐related volunteer databases and social media.

**Statement of contribution**

***What is already known on this subject***

- Having a sibling/parent with an autism/ADHD diagnosis increases likelihood of receiving diagnosis.
- A family member with autism/ADHD increases likelihood of other psychiatric conditions.
- Autism and ADHD are both associated with socio‐economic status and education.

***What the present study adds***

- Prevalence of family history of autism/ADHD is 6% but varies by ascertainment type.
- Prevalence is higher when participants were recruited through online pool versus through babylabs.
- Results should be considered when planning recruitment strategies for studies.

## BACKGROUND

Autism Spectrum Condition (hereafter ‘autism’) and Attention Deficit Hyperactivity Disorder (ADHD) are highly heritable, neurodevelopmental conditions. The prevalence of autism is around 1% (Fombonne et al., 2021), with similar rates reported amongst UK school‐aged children (Roman‐Urrestarazu et al., 2021). The heritability of autism means that children with a parent or sibling with a diagnosis have a higher likelihood of receiving a diagnosis themselves. The recurrence rate for autism is ~10% (Constantino et al., 2010; Risch et al., 2014), although rates in prospective sibling studies are somewhat higher, up to ~20% (Messinger et al., 2013; Ozonoff et al., 2011). This compares to a 0.5% prevalence in siblings with an older sibling without a diagnosis (Risch et al., 2014). Recurrence rates for half‐siblings are also increased, 5%–7% and ~2% for maternal and paternal half‐siblings respectively (Constantino et al., 2013; Risch et al., 2014).

ADHD occurs in 3%–5% (Polanczyk et al., 2007, 2015) of the population [similar amongst UK school‐aged children (Russell et al., 2019)]. Rates for ADHD are around 8–13 times higher for children with a diagnosed sibling (Chen et al., 2017; Miller et al., 2019) and 2–3 times for those with diagnosed maternal and paternal half‐siblings (Chen et al., 2017). Having a parent with a diagnosis of ADHD increases the likelihood of a diagnosis 5 times (Musser et al., 2014).

Recent meta‐analyses show the co‐occurrence of autism and ADHD is around 30%–40% (Lai et al., 2019; Rong et al., 2021), with a cohort study estimating the prevalence rate of having both conditions as around 0.73% (Ghirardi et al., 2018). Having another family member, such as a sibling, with autism also increases a person's likelihood of an ADHD diagnosis and vice versa (Odds Ratio: ~4) (Ghirardi et al., 2018; Jokiranta‐Olkoniemi et al., 2016, 2018; Miller et al., 2019) and increases the likelihood of other psychiatric conditions and developmental delays (Jokiranta‐Olkoniemi et al., 2016, 2018). Around 20% of children with an older sibling with autism but without a diagnosis themselves, show elevated autism traits, lower levels of developmental functioning (Messinger et al., 2013) or language delays (Constantino et al., 2010). Thus, having a family history (FH) of either autism or ADHD means that a child is at an increased likelihood of neurodivergent development compared to the children without a FH of autism/ADHD (Charman et al., 2023).

Studies recruiting infants to investigate general development often obtain information on diagnosed medical conditions or birth complications to exclude or control for factors which may influence the behaviour or skill being studied. However, as a diagnosis before the age of 3 years is uncommon for autism [<20% in the United Kingdom (Brett et al., 2016)] and even rarer for ADHD (Hoang et al., 2019), this is often not yet known at recruitment. Studies, including for example large longitudinal cohort studies [e.g. (Kooijman et al., 2016)], do not routinely ask about the diagnostic status of the infant's parents and siblings, even though, as outlined above, this is likely to be related to the development of the infant. To understand the scale of this issue, it is, therefore, useful to study the prevalence of autism and ADHD FH in samples participating in developmental studies and whether prevalence rates differ based on recruitment approach, which may impact the generalizability of the findings.

Some commonly used recruitment approaches for developmental psychology research include the use of volunteer databases related to universities and advertisement on social media. However, these recruitment techniques often lead to an underrepresentation of people with lower incomes or educational levels, or from minoritized ethnicities (Patel et al., 2003). More recently, crowdsourcing websites such as Amazon Mechanical Turk and Prolific are used to run online questionnaires and experiments. These platforms have the benefit that they reach a wider pool of participants (Palan & Schitter, 2018), potentially leading to samples more representative of the general population. An online platform for developmental research, Lookit (recently merged with ‘Children Helping Science’), is reported to reach a sample more representative of the US population (Scott & Schulz, 2017). We are not aware of any study comparing the demographics of samples recruited through crowdsourcing versus more traditional approaches in infant research within the United Kingdom.

The demographic characteristics of samples which potentially differ by recruitment strategy, such as socio‐economic status [SES; often measured through parental educational attainment and household income (Davis‐Kean et al., 2021)], may be differentially associated with FH. SES has been more broadly associated with more mental health problems in children (Reiss, 2013). Lower SES may also be associated with higher rates of intellectual disability amongst those with a neurodevelopmental condition (Delobel‐Ayoub et al., 2015). Co‐occurring conditions potentially lead to greater difficulties experienced by these families. In conjunction with less readily available support in the direct environment this may make the requirement of a diagnosis higher in order to access the right support. On the other hand, lower SES may be associated with no or delayed diagnoses due to reduced access to assessment and diagnostic services (Thomas et al., 2012), as these tend to depend on better financial resources (e.g. bypass waiting list via private assessments). This could lead to lower reported prevalence of FH amongst lower SES families (Fadus et al., 2020).

Consistent with this, previous studies report associations between SES and autism in both directions. In a genome‐wide association study (GWAS), genetic liability for higher educational attainment is associated with an increased likelihood of autism when considering cognitive ability (Dardani et al., 2021). Similarly, in UK‐based population studies, higher parental education is associated with higher rates of autism (Baird et al., 2006; Kelly et al., 2019). In contrast, a UK population sample of children in state‐funded education shows that children with autism are 1.6 times more likely to have social disadvantages, measured by their eligibility for free school meals (Roman‐Urrestarazu et al., 2021). The reported differences between studies may be due to differences in ascertainment (Rai et al., 2012).

Findings regarding ADHD more consistently show an association between lower SES and higher rates of ADHD. Lower parental socio‐economic position is associated with more ADHD diagnoses (Hegelund et al., 2019) and those with ADHD are 6 times more often from lower, compared to higher income families (Rowland et al., 2018). Similarly, those with a genetic liability for higher educational attainment are less likely to have ADHD (Dardani et al., 2021). A recent meta‐analysis has also found those from low SES backgrounds to be twice as likely to have ADHD (Russell et al., 2016).

Thus, whereas associations between SES and autism have been found in both directions [e.g. (Dardani et al., 2021) higher SES and higher autism, (Roman‐Urrestarazu et al., 2021) opposite], ADHD has more consistently been related to lower SES. These associations between SES and diagnosed neurodevelopmental conditions are likely the result of a complex interaction between genetic influences and the environment (Russell et al., 2014), including socio‐economic circumstances and social bias (Begeer et al., 2009; Fadus et al., 2020). More research is needed to investigate the potential differential association between FH and SES. The current study including multiple samples, obtained through different recruitment sources and with likely varying levels of SES, can contribute to this literature.

The current exploratory study aims to identify the rates of FH of autism and ADHD amongst infants taking part in developmental studies and investigate whether FH varies by ascertainment and with SES. As we are expecting recruitment approach to impact the demographics and potentially the FH of the sample, we also look at the association between FH and SES across these general samples and see if a differential association exists. Understanding the rate of FH of neurodevelopmental conditions samples recruited via university babylabs versus crowdsourcing platforms will be helpful in determining the generalizability of results of studies using these approaches. Equally, knowing whether or not to anticipate a high rate of FH based on the planned recruitment approach, gives researchers the opportunity to collect data that will enable them to account for FH in the study design and analytic approach. To address these questions, FH information was collected from three UK‐based samples, recruited via commonly used approaches, e.g., hospitals, online advertising and crowdsourcing platforms. These samples were not recruited based on the absence or presence of FH of autism or ADHD.

## METHODS

### Samples

#### Oxford EF study (OEEF)—Lab‐based via University‐led recruitment (OEEF‐Lab/Uni)

Families were recruited for the pilot (*N* = 136; collected from Autumn 2017 to Spring 2019) or main phase (*N* = 179; collected from Spring 2019 to Autumn 2020) of a UK‐based in‐person study about the development of executive functions (~10 per cent of families provided questionnaire data only due to lockdown restrictions). Respondents were recruited via the Oxford University babylab social media pages and volunteer database. Participants reported they heard of the babylab via social media (20%), word of mouth (21%), maternity wing of hospital (40%) or other sources including links with university and previous studies (19%). Thirteen participants (pilot: *n* = 9, main: *n* = 4) did not complete the questionnaire and 2 (pilot: *n* = 1, main: *n* = 1) questionnaires had errors (i.e., indicated family history but did not identify any family member) and were excluded. The final OEEF‐Lab/Uni sample consisted of 300 participants [*M*_age_ = 12.50, *SD* = 6.08, range: 9–38 months; 162 males (54%)]. Ethics approval was granted by University of Oxford Medical Sciences Interdivisional Research Ethics Committee; Ref. No. R39996/RE001 and R57972/RE001. Parents provided informed consent on behalf of themselves and their infants. At the end of each in‐person visit, families were given a branded gift costing under £5. Experimental data from these participants have been previously reported by Fiske et al. (2022), Hendry et al. (2021), Hendry and Holmboe (2021) and Lui et al. (2021).

#### Social distancing and development study (SDDS)—Online via University‐led recruitment (SDDS‐Online/Uni)

Participants were recruited in Spring 2020 from across the United Kingdom to take part in an online questionnaire study on language and cognitive development during the pandemic. Advertisements were shared online on research‐related websites and social media groups and via the Oxford Brookes babylab volunteer database. Recruitment sources for the database include word of mouth, free and paying baby groups, family events, in‐hospital recruitment and social media. Parents provided information on socio‐demographic characteristics at the first testing point. Family history data were collected during the third follow‐up (November–December 2020) in which 253 families took part [*M*_age_ = 26.62, *SD* = 7.35, range: 14–46 months; 125 males (49%)], from an original 862 who had provided socio‐demographic data. Ethics approval was granted by Oxford Brookes University Research Ethics Committee; Ref. No. 20023. Parents provided informed consent on behalf of themselves and their children. Full study details can be found in Davies et al. (2021). On completion of the first two data collection points, families were given a £30 Amazon voucher. On completion of the third data collection point, families were given a £5 Amazon voucher.

#### Prolific—Online via crowdsourcing platform (Prolific‐Online/Crowd)

Participants were recruited via Prolific (https://www.prolific.co/), an online participant pool platform for research studies, in May 2021 to take part in a 1‐min online screener questionnaire (Mdn = 55 s; min: 20 s, max:10 min 22 s) about the early development of thinking and regulation skills. Prolific pre‐screeners were selected to only include participants with a child born in 2019 (i.e., 17–28 months) and a UK nationality. Ethics approval was granted by University of Oxford Medical Sciences Interdivisional Research Ethics Committee; Ref. No. R64473. Of the 562 entries, 26 had entered the study twice, of which 22 were recorded by Prolific as spam and 4 were manually identified. 18 participants did not complete the study or revoked their consent and 3 had errors (i.e., indicated family history but did not identify any family member) and were excluded. Of the remaining 515 participants, 12 participants indicated they lived outside of the United Kingdom and for 1 participant, these data were missing. Given that participants in the other samples all lived in the United Kingdom, these were not included. The final sample consisted of 502 participants. All participants were compensated £0.50 for their time.

Part of the Prolific‐Online/Crowd sample was followed up (Prolific‐Online/Crowd‐FU) to collect more data on socio‐demographic characteristics and the child's cognitive and social development. All families who indicated that they had a diagnosed or suspected FH of autism or ADHD were invited to participate between June and August 2021 (*N* = 48). Families who did not indicate an FH of autism or ADHD were followed up in Sep 2021 (*N* = 44). Respondents in either group were compensated £5 for their time. The main Prolific‐Online/Crowd sample was used to look at FH rate, whereas the Prolific‐Online/Crowd‐FU sample was used to look at the association between FH and SES.

### Sample characterization

The OEEF‐Lab/Uni, SDDS‐Online/Uni and Prolific‐Online/Crowd‐FU samples were asked to report on the number of children in the family, their household income and highest level of education for both mother and father. For the main Prolific‐Online/Crowd sample, these data were only available at a sample level.

Data from the samples were merged based on the highest‐level data available in all three datasets to create equivalent categories. The number of categories was reduced to ensure large enough groups. Three education categories were created: A‐level or below (including vocational courses), Undergraduate degree and Postgraduate or equivalent degree (includes advanced professional qualifications). For the OEEF‐Lab/Uni and SDDS‐Online/Uni samples, data from both parents was available. These data were merged using the highest level of education of both parents in the analyses. Household income was recoded into low‐, below‐median‐ and above‐median‐income groups. The median equivalised income was £30,500 in the United Kingdom in 2020 (Office for National Statistics, 22 March 2022). This income is adjusted for the household composition using the OECD‐modified scale (Hagenaars et al., 1994), based on the number of adults and children (for our sample, this number is 1.96 based on OEEF‐Lab/Uni, SDDS‐Online/Uni and Prolific‐Online/Crowd‐FU samples). Taking this adjustment into consideration, an income of £60,000 for our families is roughly equivalent to the UK median. As very few families had an income below £20,000 and we have limited detail over £70K, we categorized <£30,000 as low, £30,000–£60,000 below median and >£60,000 as above median.

### Characterizing sample demographic differences

Details on the number of children and SES indices are given in Table 1. More detailed sample characterization data, including Prolific‐Online/Crowd‐FU sample, can be found in the appendix, Appendix Table S1. There was a significant difference between the three main samples based on the number of siblings [*χ* ^2^(2) = 25.80, *p* < .001], education level [*χ* ^2^(4) = 202.57, *p* < .001] and household income [*χ* ^2^(4) = 119.65, *p* < .001]. Inspecting the adjusted residuals (>|2|) for the number of children, there were relatively more families with one child in the OEEF‐Lab/Uni sample, whereas the Prolific‐Online/Crowd sample had relatively more families with multiple children.

**TABLE 1:** Sample descriptives.

	OEEF‐Lab/Uni (*N* = 300)	SDDS‐Online/Uni (*N* = 253)	Prolific‐Online/Crowd (*N* = 502)	*χ* ^2^ (df)
Number of siblings				
No siblings	210 (70.0%)^a^	163 (64.4%)	234 (52.2%)^b^	25.80 (2), *p* < .001
1+ siblings	90 (30.0%)^b^	90 (35.6%)	214 (47.8%)^a^	
Unknown	—	—	54	
Education level				
A‐level or below	23 (7.7%)^b^	20 (7.9%)^b^	150 (39.9%)^a^	202.57 (4), *p* < .001
Undergraduate	86 (28.8%)	74 (29.3%)	146 (38.8%)^a^	
Postgraduate or equivalent	190 (63.5%)^a^	159 (62.8%)^a^	80 (21.3%)^b^	
Unknown	1	—	126	
Household income				
Low: 0–30 K	12 (5.0%)^b^	38 (15.0%)	110 (26.0%)^a^	119.65 (4), *p* < .001
Below‐median: 30–60 K	87 (35.9%)^b^	103 (40.7%)	227 (53.7%)^a^	
Above‐median: 60 K+	143 (59.1%)^a^	112 (44.3%)^a^	86 (20.2%)^b^	
Unknown	58	—	79	

*Note*: Percentages shown exclude cases unknown.

^a^ Adjusted residuals above 2.

^b^ Adjusted residual below −2.

For education level, the OEEF‐Lab/Uni and SDDS‐Online/Uni samples had relatively (compared with the overall sample) fewer participants with an A‐level or below, and more with a Postgraduate or equivalent level. The education qualification level of the OEEF‐Lab/Uni and SDDS‐Online/Uni samples was higher compared to that of the UK population between 25 and 49 years of age (54% of people with an educational qualification above A‐level) (UK Government, 2021). The Prolific‐Online/Crowd dataset had relatively more participants with an A‐level or below, or an Undergraduate degree, and fewer with a Postgraduate or equivalent level compared with the overall sample but was comparable to that of the general UK population.

Regarding household income, the OEEF‐Lab/Uni and SDDS‐Online/Uni samples had relatively more participants in the above‐median category; and the OEEF‐Lab/Uni sample also had relatively fewer in the low or below‐median category. The Prolific‐Online/Crowd dataset had relatively more participants in the low or below‐median income categories and fewer in above‐median. Overall, participants in OEEF‐Lab/Uni more often earned above, SDDS‐Online/Uni earned just below and most people in Prolific‐Online/Crowd earned below the median UK income.

In sum, the OEEF‐Lab/Uni and SDDS‐Online/Uni samples were of higher SES than the Prolific‐Online/Crowd sample and overall had an above‐average SES, whereas Prolific‐Online/Crowd had a lower SES compared to the general UK population. The Prolific‐Online/Crowd‐FU sample differed from the overall Prolific‐Online/Crowd sample in the number of siblings [*χ* ^2^(1) = 32.73, *p* < .001; more siblings in 3‐Online/Crowd‐FU sample], but not in education level or household income (see Appendix S1 for Prolific‐Online/Crowd‐FU details).

### Family history of autism and ADHD

In all three studies, parents were asked to report whether any immediate blood relatives of the target child had a confirmed diagnosis of autism, ADHD or both. If so, they specified how many and which family member(s), (i.e. father, mother, brother, sister, half‐brother or ‐sister, separately for maternal and paternal side). Exact phrasing can be found in the Appendix S1. In the Prolific‐Online/Crowd dataset, parents were additionally asked to report the number of suspected cases of autism or ADHD (suspected FH). For the OEEF‐Lab/Uni and SDDS‐Online/Uni datasets, diagnosed FH data were also collected on the wider family, including grandparents, aunts or uncles and cousins (extended FH). The results and discussion on suspected FH and extended FH can be found in the Appendix S1.

## RESULTS

All analyses were performed in Stata 17.0. All post‐hoc results are reported using the Holm‐Bonferroni method to adjust for multiple comparisons.

### Rate of family history of autism and ADHD

Of the 1034 (excluding 21 families reporting ‘do not know’) families who reported on family history, 5.8% reported that one or more family members had diagnosed autism, ADHD or both (Autism: 1.9%; ADHD: 3.5%; Both: 0.4% see Table 2). Of the 17 children in the OEEF‐Lab/Uni and SDDS‐Online/Uni samples with a FH 8 (47%) were males.

**TABLE 2:** Family history.

	OEEF‐Lab/Uni (*N* = 300)	SDDS‐Online/Uni (*N* = 253)	Prolific‐Online/Crowd (*N* = 502)	Full sample (*N* = 1055)	Fisher's exact (*p*)^a^
Family history					
Autism (‘FH‐autism’)	3 (1.0%)	2 (0.8%)	15 (3.0%)	20 (1.9%)	.027
ADHD (‘FH‐ADHD’)	7 (2.4%)	4 (1.6%)*	25 (5.1%)*	36 (3.5%)	.013
Autism & ADHD (‘FH‐both’)	1 (0.4%)	—	3 (0.6%)	4 (0.4%)	
Autism and/or ADHD (‘FH‐any’)	11 (3.8%)	6 (2.4%)*	43 (8.7%)*	60 (5.8%)	.001
No Autism or ADHD diagnosis (‘No‐FH’)	279 (96.2%)	243 (97.6%)	452 (91.3%)	974 (94.2%)	
Do not know	10	4	7	21	

*Note*: Percentages shown exclude ‘do not know’.

^a^ Fisher's exact test was used to compare the three studies on FH‐autism/both versus no‐FH‐autism; FH‐ADHD/both versus no‐FH‐ADHD and FH‐any versus No‐FH. Differences between samples are indicated with an asterisk (*) if significant after Holm‐Bonferroni correction.

Exploring the rate further, of those families with a diagnosed FH, the majority identified one person with a diagnosis of autism (87.5%) or ADHD (85%). For autism, 2 families reported autism in two family members and 1 family reported autism in three family members. For ADHD, 5 families reported ADHD in two family members and 1 in three family members. Of the individual family members with a diagnosis, 61% of those with autism and 77% of those with ADHD were male.

The overall rate was calculated based on the total number of first‐degree family members within each family with a diagnosis reported in the OEEF‐Lab/Uni and SDDS‐Online/Uni samples (total number of first‐degree family members was not available for the Prolific‐Online/Crowd sample). Considering the total number of first‐degree family members in OEEF‐Lab/Uni and SDDS‐Online/Uni samples (*N* = 1289, excluding the target child), the rate of autism was 0.6% (*N* = 8; CI: 0.2%–1.2%) and 0.9% for ADHD (*N* = 12; CI: 0.5%–1.6%). Rates amongst parents only (including all three studies: *N* = 2068) is 0.8% (CI: 0.4%–1.3%) for autism and 1.5% (CI: 1.0%–2.1%) for ADHD.

*Suspected cases*: Including families that indicated they suspected a diagnosis of autism, ADHD or both in one or multiple family members, the total rate of FH in Prolific‐Online/Crowd was 17% (35 FH‐autism, 36 FH‐ADHD and 13 FH‐both). Of the FH‐any families, 35% suspected a diagnosis of autism, ADHD or both amongst their family members. Amongst no‐FH families, this was 9%. Individuals with suspected diagnoses were more often male (autism: 62%; ADHD: 61%). Full details and additional analyses can be found in the Appendix (Table S2).

### Family history by ascertainment

The rate of FH in the core family was compared between the three samples using Fisher's exact test [given that some of the cells had an expected count below 5 (Kim, 2017)]. For post‐hoc comparisons, the p‐value was adjusted using the Holm‐Bonferroni method. The analyses for FH‐autism and FH‐ADHD also include those children who have a FH for both and was compared to those without a FH of autism or ADHD, respectively.

FH‐autism (*p* = .027) and FH‐ADHD (*p* = .013) rates differed between samples. Post‐hoc comparisons for FH‐autism showed that none of the contrasts were significant (SDDS‐Online/Uni vs Prolific‐Online/Crowd: *p* = .028, which is larger than the corrected p‐value of .017). Rate of FH‐ADHD was higher in Prolific‐Online/Crowd (5.1%) compared to SDDS‐Online/Uni (1.6%; *p* = .012; OR = 3.67 [1.27, 10.59]). None of the other comparisons were significant.

Results were similar for FH‐any (Fisher's exact: *p* = .001); the rate of FH‐any was higher in the Prolific‐Online/Crowd compared to the SDDS‐Online/Uni (*p* = .001; OR = 3.85 [1.62, 9.18]) and OEEF‐Lab/Uni (*p* = .008; OR = 2.41 [1.22, 4.76]) samples. This difference could be due to the Prolific‐Online/Crowd sample including more siblings, thus more chance of receiving a diagnosis in a family member. Because we do not have details available on the number of siblings of each individual family in the Prolific‐Online/Crowd sample, we repeated the analysis including only parental diagnosis to make the samples more comparable. The Fisher's exact test was repeated including only parental diagnosis of autism or ADHD. No differences between samples were found for either FH‐autism or FH‐ADHD, but results for FH‐any were similar to when all family members were included (*p* = .014), with a significant difference between the Prolific‐Online/Crowd (6.1%) sample and SDDS‐Online/Uni (2.0%; *p* = .016; OR = 3.15 [1.21, 8.22]) sample.

### Associations between FH and indices of SES

Across the sample (OEEF‐Lab/Uni, SDDS‐Online/Uni and Prolific‐Online/Crowd‐FU), Fisher's exact tests showed no significant association between FH‐autism and household income, but there was a significant association with education level (*p* < .001). Pairwise comparisons showed that compared to the families without a FH‐autism, there were relatively more FH‐autism families with A‐level or below than an undergraduate (*p* = .014; OR = 3.49 [1.28, 9.53]) or postgraduate or equivalent degree (*p* < .001; OR = 15.99 [4.30, 59.52]), and more FH‐autism families with an undergraduate versus a postgraduate or equivalent degree (*p* = .037; OR = 4.57 [1.17, 17.91]) (see Appendix Table S4 for number of families per category).

There was a significant association between FH‐ADHD and income (*p* < .001). Compared to the families without FH‐ADHD, there were relatively more FH‐ADHD families with a low than an above‐median income (*p* = .001; OR = 8.70 [2.54, 29.84]). For education level (*p* = .002), relatively more families with FH‐ADHD fell in the middle than the high education group compared to the no‐FH families (*p* = .001; OR = 4.72 [1.78, 12.50]) (also see Appendix Table S4).

## DISCUSSION

This study aimed to understand the rates of elevated likelihood of neurodivergent development, by virtue of a FH of autism or ADHD, amongst infants taking part in developmental studies and to investigate whether FH of autism or ADHD is associated with SES. A particular strength of this study is the inclusion of three different samples which used a range of recruitment sources and study delivery approaches (OEEF‐Lab/Uni: lab‐based study recruiting via a university volunteer database and social media; SDDS‐Online/Uni: online study recruiting via a university volunteer database and social media; Prolific‐Online/Crowd: online study recruiting via an online crowdsourcing platform) common in developmental research. In all three studies, parents reported diagnoses of autism, ADHD or both in first‐degree family members of the target child. OEEF‐Lab/Uni and SDDS‐Online/Uni also asked about extended family, and the Prolific‐Online/Crowd sample reported on suspected cases in first‐degree family members (see Appendix S1). It should be noted that the sample size is too small to obtain a true prevalence of FH in the UK population.

Across the combined samples, 6% of families reported a FH of autism, ADHD or both. As expected, more males (dads, (half)brothers) have received an autism or ADHD diagnosis compared to females [mums, half(sisters)] (Posserud et al., 2021). The percentage of families with suspected FH of autism or ADHD (recruited via the crowdsourcing platform only) was 17%. As siblings of those with autism or ADHD are at an increased likelihood of a diagnosis themselves (Miller et al., 2019) and family members show higher levels of sub‐clinical traits (Messinger et al., 2013), psychiatric conditions and more general developmental delay (Charman et al., 2023; Jokiranta‐Olkoniemi et al., 2016, 2018), these numbers suggest that a reasonable proportion of children in these studies may be expected to be neurodivergent. Due to the high heritability of neurodevelopmental conditions, considering family history is an important aspect of the diagnostic process of autism and ADHD (National Institute for Health and Care Excellence, 2017, 2019), but family history is not often considered in research. Developmental researchers, after excluding for rare conditions (e.g., genetic syndromes), prematurity or medical problems at birth, and other serious health events, often describe their samples of ‘typically developing’: our data indicate that in fact such samples are likely to be neurodiverse. To better understand the extent to which this is the case, it is recommended that researchers collect FH data as part of the demographic profile of their participants, especially when researching topics that overlap with autism or ADHD traits, such as social development and attention. Equally, in studies comparing autism or ADHD samples with a control sample, researchers should consider excluding control children with a FH of neurodevelopmental conditions. It should be noted that diagnostic status of the target child was not included in the current study, therefore we cannot say how many of the children in these samples are neurodivergent. However, considering the age of the target children (9 to 46 months) and the average age of autism and ADHD diagnosis (Brett et al., 2016; Hoang et al., 2019) it is unlikely that including diagnostic data would lead to an accurate picture.

Amongst the families without a confirmed family history of autism/ADHD, 9% suspected a family member had autism, ADHD or both, versus 35% of those with a FH of either. It is likely that parents with a family member already diagnosed are more aware of the traits and are thus more likely to suspect autism/ADHD prior to a formal diagnosis. During the diagnostic process, parental concerns about autism increase the sensitivity of diagnostic instruments, more frequently detecting those with autism (Havdahl et al., 2017). Families who are more aware of autism traits because of another family member with a diagnosis, may, therefore, also be more likely to receive a diagnosis for another child.

There are several possible reasons why neurodivergence may be suspected and indeed present but not accompanied by a diagnosis, including lack of access to diagnostic services, stigma around labels and people not seeing the value of a confirmed diagnosis. If the number of families with a FH of autism/ADHD is indeed closer to the suspected rather than confirmed proportion reported, this has a bearing on whether and how researchers screen for FH. Thus, this large discrepancy between confirmed and suspected autism/ADHD merits further investigation.

The data also indicate that the neurodiversity of a sample may be influenced by the recruitment strategy. More families in the Prolific‐Online/Crowd sample reported having a family member with diagnosed ADHD, compared to the SDDS‐Online/Uni sample. Similar trends for autism did not survive correction for multiple comparison. No differences were found between the OEEF‐Lab/Uni and the other two samples. Because the Prolific‐Online/Crowd respondents also reported that the infant had more siblings, thus more potential family members in which a diagnosis can be present, the analyses were repeated including only parents. The effects of FH‐ADHD and FH‐autism were no longer significant; however, parents did still report any FH more often in Prolific‐Online/Crowd compared to SDDS‐Online/Uni. Thus, the sample obtained via an online recruitment platform showed the highest FH of autism/ADHD, with 9% with a diagnosed family member. When including suspected FH of autism/ADHD in family members, the rate was 17% (see Appendix S1). In terms of SES, the sample recruited through Prolific‐Online/Crowd included more low SES families, compared to those recruited through university volunteer databases or social media accounts. These sample differences in SES and neurodiversity should be considered by researchers when choosing their recruitment strategies.

Our results show that using crowdsourcing platforms to recruit parents for completing studies online could be a good resource to reach populations that may be harder to reach through university babylabs, especially those of a lower SES. Previous studies have reported on the reliability of data collected through crowdsourcing platforms, especially Prolific (Palan & Schitter, 2018; Peer et al., 2017), but verifying the identity of the respondents is more difficult and it is suggested to include attention checks to examine the respondent's engagement (Newman et al., 2021), which we did not include in the current study. The total time of our survey was, however, very brief (Median = 55s) thus it is unlikely that participants lost attention during the survey.

In recent years there have been major advances in the types of infant studies that can be administered online, e.g. using the Lookit (now Children Helping Science) platform (Scott & Schulz, 2017). Gaze data can be collected from child participants remotely, leading to results comparable to those from laboratory studies (Scott et al., 2017). However, there are constraints on the data quality and the type of tests that can be performed, and remote neuroimaging studies are not yet feasible. Nevertheless, there may be particular advantages to online data collection of questionnaires: Online surveys are more impersonal than visits to a research laboratory, which may reduce the implicit pressure to give socially desirable responses (Bartneck et al., 2015). However, a meta‐analysis study showed no differences in socially desirable answers between surveys done online or in‐person (Dodou & de Winter, 2014). Thus, although crowdsourcing platforms have some limitations, they can be used to complement in‐person research to include a more diverse population in developmental research.

The overall rate of autism (0.6%) and ADHD (0.9%) amongst family members of the infant (not including the target infant) in OEEF‐Lab/Uni and SDDS‐Online/Uni were lower compared to those observed in two large UK‐based cohort studies (Roman‐Urrestarazu et al., 2021; Russell et al., 2019). This could indicate that families with a FH of autism/ADHD are less likely to participate in research studies on early child development. However, the target infants were between 9 and 46 months when their parents completed the questionnaire and with the average age gap in the United Kingdom between the first and second child being 2.3 years, it is likely that older siblings were still too young to have received a diagnosis, particularly for ADHD, which is usually diagnosed in mid‐childhood (Hoang et al., 2019; Rocco et al., 2021). Thus it is likely that the rate of confirmed FH of autism/ADHD in our sample will increase over time. Looking at rates amongst parents only (autism: 0.8%; ADHD: 1.5%), these were similar to the adult population in the United Kingdom for autism [1.1% (Brugha et al., 2016)], but below worldwide adult population estimates for ADHD [2.5% (Simon et al., 2009)], which could be due to underdiagnosis.

The overall number of families with both an autism and ADHD diagnosis across our samples was low (of the any‐FH families, only 6.7% had both autism and ADHD in the family), compared to data from a national survey showing that 12% of children with ADHD are also diagnosed with autism (Zablotsky et al., 2020). A meta‐analysis including 63 studies found ~40% of those with autism have ADHD (Rong et al., 2021). These lower rates may be due to the age of the siblings, or to changes in diagnostic practice: Under DSM‐4 (American Psychiatric Association, 2000), used until the publication of DSM‐5 (American Psychiatric Association, 2013) in 2013, individuals could only be diagnosed with one of the two conditions, such that parents who received a diagnosis before the use of DSM‐5 would not have been given a dual diagnosis, even if presenting with both autism and ADHD.

Looking at the association between a FH of autism and SES, it was found that parents in the FH‐autism group had a lower education level than parents without a FH of autism. An association between SES and FH‐ADHD was also found, showing fewer FH‐ADHD families in the high‐income and education level groups compared to the no‐FH group. This is largely in line with previous studies showing an association between lower SES and autism within a large Danish cohort study (Hegelund et al., 2019), and using data from large pupil databases in the United Kingdom (Roman‐Urrestarazu et al., 2021) and with ADHD in the United States (Rowland et al., 2018). We did not find an association between higher education attainment and autism found in previous studies [e.g. a GWAS study (Dardani et al., 2021)]. Considering the broad spectrum of autism, it is possible that associations with SES exist in both directions. For example, autism may more commonly co‐occur with intellectual disability amongst low SES families (Delobel‐Ayoub et al., 2015), potentially leading to more difficulties and therefore families may be more likely to seek a diagnosis. Other the other hand, families with high SES may have more access to diagnostic services due to better financial resources. We did not find evidence for the latter within our UK‐based samples. Moreover, we did not find evidence that suspected cases differed in SES from those with a confirmed diagnosis (see Appendix S1).

When asking about household income, we did not specify whether this included any benefits received. Moreover, our household income data were not detailed enough to adjust for household composition at an individual level. Considering potential differences in household size between FH and no‐FH families, due to for example higher rates of divorce amongst families with autism (Hoover & Kaufman, 2018) or ADHD (Insa Pineda et al., 2020) [although also see (Freedman et al., 2012)], correcting for household composition could have given more precise data regarding SES. Indeed, within our datasets, one‐adult households were more common amongst FH‐any families, compared to no‐FH families (see Appendix S1). However, looking at a sample level, it is unlikely this would have changed the direction of the results. OEEF‐Lab/Uni and SDDS‐Online/Uni should have been adjusted by 1.9 versus 2.2 for Prolific‐Online/Crowd. Thus, the discrepancy in income levels between Prolific‐Online/Crowd versus OEEF‐Lab/Uni and SDDS‐Online/Uni would be even larger than reported if adjusted.

From this cross‐sectional study, we cannot comment on the direction of causality between SES and rates of FH of autism/ADHD. However, longitudinal studies looking at this association have shown that poverty in infancy is related to an increased likelihood of ADHD diagnosis at age seven (Russell et al., 2015). These findings indicate that FH of autism/ADHD may need to be taken into account when interpreting data indicating that lower SES is associated with developmental delays, lower academic achievement (Brooks‐Gunn & Duncan, 1997) and lower executive function (Lawson et al., 2018).

## CONCLUSION

In three samples of infants of the type commonly recruited for developmental studies, it was found that the rates of a FH of autism/ADHD ranged between 2% and 9%. The rates of FH‐autism or FH‐ADHD was higher when participants were recruited via an online crowdsourcing platform (comprising a more socio‐economically diverse sample), compared to when recruited through university volunteer databases and social media (which were skewed to higher SES). The prevalence of FH‐autism or FH‐ADHD increased to 17% when suspected FH of autism/ADHD was also considered (in the sample including more low‐SES families). Lower parental education levels were associated with higher rates of FH‐autism and FH‐ADHD and lower family income with higher rates of FH‐ADHD. As infants with a FH of autism/ADHD may show alternative profiles of cognitive and social development compared with infants from neurotypical families, it is crucial that developmental researchers take this into account when planning how to recruit and characterize their samples.

## AUTHOR CONTRIBUTIONS

**Tessel Bazelmans:** Formal analysis; writing – original draft; writing – review and editing. **Gaia Scerif:** Supervision; writing – review and editing. **Karla Holmboe:** Funding acquisition; methodology; writing – review and editing. **Nayeli Gonzalez‐Gomez:** Methodology; funding acquisition; writing – review and editing. **Alexandra Hendry:** Conceptualization; funding acquisition; methodology; writing – review and editing.

## CONFLICT OF INTEREST STATEMENT

The authors declare no conflicts of interest.

## Supporting information


Appendix S1.

## Data Availability

The data that support the findings of this study are available on request from the corresponding author. The data are not publicly available due to privacy or ethical restrictions.
