# Supplementary material for: Rates of family history of autism and ADHD varies with recruitment approach and socio‐economic status
Source: Br J Dev Psychol. 2023 Nov 16;42(2):117–32. doi: 10.1111/bjdp.12469 (PMC11256865; doi:10.1111/bjdp.12469)
Supplement: Supplementary file 1 — Appendix S1. [file BJDP-42-117-s001.docx]

# **Appendix**

**Methods**

**Detailed sample characterisation per sample.**

For household income, the OEEF-Lab/Uni samples were asked to provide a value, the SDDS-Online/Uni and Prolific-Online/Crowd samples had household income information available based on 7 categories (ranging from: 1. £0 - £20k to 7. £71k+). The highest level of education was asked in 6 categories for OEEF-Lab/Uni and 8 categories for SDDS-Online/Uni (up to ‘doctoral degree’).

Table A1. *Detailed description of number of children and SES per sample.*

|  | OEEF-Lab/Uni | | SDDS-Online/Uni | | Prolific-Online/Crowd | Prolific-Online/Crowd FU | |
| --- | --- | --- | --- | --- | --- | --- | --- |
|  | N = 300 | | N = 253 | | N = 502 | N=92 | |
| *Number of children* |  | |  | |  |  | |
| 1 | 210 (70%) | | 163 (64%) | | 234 (47%) | 18 (20%) | |
| 2 | 78 (26%) | | 74 (29%) | | 142 (28%) | 41 (45%) | |
| 3+ | 12 (4%) | | 16 (6%) | | 72 (14%) | 33 (36%) | |
| Unknown | - | | - | | 54 (11%) | - | |
| *Education Level* | *Mother* | *Father* | *Mother* | *Father* | *Parent* | *Mother* | *Father* |
| GCSE or below | 13  (4%) | 39  (13%) | 3  (1%) | 15  (6%) | 40  (8%) | 26 (28%) | 36  (39%) |
| A-level, vocational degree or equivalent | 20  (7%) | 23  (8%) | 25  (10%) | 53 (21%) | 110  (22%) | 20  (22%) | 14  (15%) |
| Undergraduate | 105 (35%) | 102 (34%) | 93  (37%) | 79 (31%) | 146  (29%) | 35 (38%) | 18  (20%) |
| Postgraduate or equivalent | 157 (52%) | 118 (39%) | 132 (52%) | 97 (38%) | 80  (16%) | 11 (12%) | 14  (15%) |
| Unknown | 5  (2%) | 18  (6%) | - | 9  (4%) | 126  (25%) | - | 10  (11%) |
| *Household income* |  | |  | |  |  | |
| 0-20K | 4 (1%) | | 12 (5%) | | 44 (9%) | 7 (8%) | |
| 20-30K | 8 (3%) | | 26 (10%) | | 66 (13%) | 11 (12%) | |
| 30-40K | 19 (6%) | | 30 (12%) | | 96 (19%) | 14 (15%) | |
| 40-50K | 25 (8%) | | 38 (15%) | | 78 (16%) | 14 (15%) | |
| 50-60K | 43 (14%) | | 35 (14%) | | 53 (11%) | 10 (11%) | |
| 60-70K | 52 (17%) | | 31 (12%) | | 28 (6%) | 7 (8%) | |
| 70K+ | 91 (30%) | | 81 (32%) | | 58 (12%) | 9 (10%) | |
| Unknown | 58 (19%) | | - | | 79 (16%) | 20 (22%) | |

**Family History Questions**

*OEEF-Lab/Uni & 2-Online/Uni:*

Question: Has any blood relative of the child participating in the study received a diagnosis of Attention Deficit Hyperactivity Disorder (ADHD) and/or Autism Spectrum Disorder (ASD)? Only consider family members who are biologically related to the child, including: Parent, Brother/Sister, Half-brother/half-sister, Aunt/Uncle, Cousin, Grandparent, Great aunt/uncle (i.e., mother's or father's uncle), Great grandparent. If they answered:

Yes, a family member of the child has received a diagnosis of ADHD or Yes, a family member of the child has received a diagnosis of ASD, this was followed up with (and repeated for ADHD and ASD separately if answered both with yes): Tick the box of blood relatives of the child participating in the study who have received a diagnosis of [ADHD / ASD] (that you know of). If more than one relative in the same category has received a diagnosis, put the number of relatives who have received a diagnosis in the box below. The options included: father, mother, brother, sister, and separately for father’s and mother’s side: half-brother, half-sister, uncle, aunt, male cousin, female cousin, grandfather, grandmother, great uncle, great aunt, great grandmother, great grandfather.

*Prolific-Online/Crowd:*

Question: Does your child have any immediate blood relatives with Attention Deficit Hyperactivity Disorder (ADHD)and/or autism? Only consider family members who are closely biologically related to the child, including: their parent (including yourself), their brother/sister, their half-brother/half-sister. The response options were: 1) Yes, a family member of the child has a diagnosis of ADHD, or is strongly suspected to have ADHD; 2) Yes, a family member of the child has received a diagnosis of Autism Spectrum Disorder (or a related diagnosis such as Asperger syndrome), or is strongly suspected to be autistic; 3) No, no family member of the child has ADHD or autism; 4) I don’t know. If their response was 1 or 2, this was followed up with(and repeated for ADHD and ASD separately if both 1 and 2 were ticked): Tick the box of blood relatives of the child participating in the study who has (or is suspected of having) [ADHD/ASD]. If more than one relative in the same category has received a diagnosis, please put the number of relatives who have received a diagnosis in the box below. The options included: father, mother, brother, sister, half-brother (father’s side), half-brother (mother’s side), half-sister (mother’s side), half-sister (mother’s side).

**Results**

**Prevalence of Family History in extended family and suspected cases**

In addition to prevalence of autism and/or ADHD in the immediate family, which was reported on in all three studies, the OEEF-Lab/Uni and SDDS-Online/Uni were asked about prevalence of autism and ADHD in the extended family and Prolific-Online/Crowd sample was additionally asked about any direct family members of the target child who they suspected to have either autism or ADHD, but who did not receive a diagnosis (yet). (see section ‘Family History Questions’). The prevalence rates are given below in Table A2. It should be noted that information collected on extended family is potentially incomplete, as the respondent may not be aware of diagnoses in the wider family as either this information is not shared or due to a lack of contact. Equally, due to better recognition of neurodevelopmental conditions, autism and ADHD may be underdiagnosed, especially in grand-parents of the target child.

The overall rate of families suspecting a family member to have autism, ADHD or both was 17 percent. Those families who already had a family member with a diagnosis were more likely to suspect the same diagnosis in another family member, compared to families who do not have a family member with confirmed diagnosis of autism or ADHD. These findings are in line with Miller et al. (2019) who also reported the highest rates of autism or ADHD in siblings with the same condition, lowest rates in those without a FH of autism or ADHD, and intermediate rates of autism or ADHD for those with a sibling with the other condition. The heritability of autism and ADHD means that having a FH of either condition makes it more likely that another family member will be neurodivergent, and it is also likely that parents with a family member already with a diagnosis are more aware of the symptoms and are thus more likely to suspect autism or ADHD prior to a formal diagnosis. Of note however, amongst the families without a confirmed family history of autism or ADHD, 9% suspected a family member had autism, ADHD or both. There are several possible reasons why neurodivergence may be suspected and indeed present but not accompanied by a diagnosis, including lack of access to diagnostic services, stigma around labels, and people not seeing the value of a confirmed diagnosis. If the number of families with a FH of autism or ADHD is indeed closer to the suspected rather than confirmed proportion reported, this has bearing on whether and how researchers screen for FH. Thus, this large discrepancy between confirmed and suspected autism or ADHD merits further investigation.

Table A2. *Family History including extended family and suspected cases.*

|  | OEEF-Lab/Uni | | SDDS-Online/Uni | | Prolific-Online/Crowd | | Full Sample |
| --- | --- | --- | --- | --- | --- | --- | --- |
|  | (N = 300) | | (N = 253) | | (N = 502) | | (N = 1055) |
| *Family History* | *First-degree*  *Diagnosed* | *Extended†*  *Diagnosed* | *First-degree*  *Diagnosed* | *Extended†*  *Diagnosed* | *First-degree Diagnosed* | *First-degree Suspected‡* | *First-degree Diagnosed* |
| Autism (‘FH-autism’) | 3 (1.0%) | 15 (5.2%) | 2 (0.8%) | 24 (9.6%) | 15 (3.0%) | 35 (7.1%) | 20 (1.9%) |
| ADHD (‘FH-ADHD’) | 7 (2.4%) | 13 (4.5%) | 4 (1.6%) | 8 (3.2%) | 25 (5.1%) | 36 (7.3%) | 36 (3.5%) |
| Autism & ADHD | 1 (0.3%) | 9 (3.1%) | - | 3 (1.2%) | 3 (0.6%) | 13 (2.6%) | 4 (0.4%) |
| Autism and/or ADHD  (‘FH-any’) | 11 (3.8%) | 37 (12.8%) | 6 (2.4%) | 35 (14.1%) | 43 (8.7%) | 84 (17.0%) | 60 (5.8%) |
| No Autism or ADHD diagnosis (‘No-FH’) | 279 (96.2%) | 253 (87.2%) | 243 (97.6%) | 214 (85.9%) | 452 (91.3%) | 411 (83.0%) | 974 (94.2%) |
| Don’t know | 10 | 10 | 4 | 4 | 7 | 7 | 21 |
| Note. Percentages shown exclude ‘don’t know’.  † Includes first-degree and extended family (grandparents, aunts, uncles and cousins)  ‡ Includes both diagnosed and suspected cases | | | | | | |  |

**Diagnosed versus Suspected in Prolific-Online/Crowd sample**

The overall rate of families with a family member either diagnosed or suspected autism and/or ADHD was 17 percent (see Table A2). Amongst the no-FH families (N=452), 41 families indicated they suspected one or more family members had autism (22; 4.9%), ADHD (13; 2.9%) or both (6; 1.3%). Of the 18 diagnosed FH-autism families, six (33%) suspected one or more additional family members to also have autism, and 2 (11%) suspected ADHD. Of the 28 families with diagnosed FH-ADHD, 7 (25%) suspected one or more additional family members had ADHD, and 2 (7%) suspected autism.

Fisher’s exact tests looking at the association between the three diagnosed FH-groups (FH-autism, FH-ADHD, no-FH) and suspected versus not suspected cases were significant for both suspected-FH-autism (*p*=.002) and suspected-FH-ADHD (*p*<.001). Follow-up pairwise tests showed that suspected-FH-autism was more common in the FH-autism group compared to the no-FH group (*p*=.001; OR = 7.57 [2.64, 21.68]), and suspected-FH-ADHD was more common in the FH-ADHD group compared to the no-FH group (*p*<.001; OR = 7.60 [2.88, 20.06]). No other contrasts were significant when considering multiple comparisons (all *p*’s>.042).

*Sibling versus parent with suspected diagnosis.* We compared how many of the suspected cases were siblings versus parents (see Table SM3). For autism, 20 families with no-diagnosed-FH indicated they suspected one or both parents and 13 suspected one or more siblings to have a diagnosis of autism. Of FH-any families, 6 indicated they suspected one or more parents and 3 families suspected one or more siblings to have autism. For ADHD, 14 families with no-diagnosed-FH indicated they suspected one or both parents and 8 suspected one or more siblings to have ADHD. Of families with FH-any, 6 suspected one or more parents and 4 suspected one or more siblings to have ADHD.

Using Fisher’s exact tests, no associations were found between FH-group (no-FH versus FH-any) and family member for either autism or ADHD (*p* = 1) for suspected cases. These results suggest that the relative number of suspected cases in parents versus siblings did not differ between families who did or did not have a diagnosed family member already. Thus, those with a FH suspected a diagnosis in parents versus siblings as often as those families without a FH.

Table A3. *Suspected autism and ADHD in parents and siblings amongst FH-groups.*

|  | Autism | | | | ADHD | | | |
| --- | --- | --- | --- | --- | --- | --- | --- | --- |
|  | FH-autism | FH-ADHD | FH-both | No FH | FH-autism | FH-ADHD | FH-both | No FH |
| Parent | 3 | 2 | 1 | 20 | 1 | 4 | 1 | 14 |
| Sibling | 3 | - | - | 13 | 1 | 3 | - | 8 |

**Associations between FH and indices of SES**

Table A4. *Number of families with/without FH-autism or FH-ADHD per education level and household income.*

|  | Autism | | ADHD | |  |
| --- | --- | --- | --- | --- | --- |
|  | FH-autism | No FH-autism | FH-ADHD | No FH-ADHD |  |
| *Education Level* | | | | | |
| A-level or below | 10 | 74 | 5 | 79 |  |
| Undergraduate | 7 | 181 | 14 | 174 |  |
| Postgraduate or equivalent | 3 | 355 | 6 | 352 |  |
| *Household income* | | | | | |
| Low: 0-30K | 3 | 65 | 8 | 60 |  |
| Below-median: 30-60K | 8 | 215 | 9 | 214 |  |
| Above-median: 60K+ | 3 | 262 | 4 | 261 |  |

**Association with SES and suspected versus diagnosed cases.**

No association was found between suspected versus diagnosed FH and household income (*p*=.813). The association with education level was *p*=.050; post-hoc analysis did not show any significant association when considering multiple comparisons. Some studies have reported an association between age at diagnosis and SES (Hosozawa et al., 2020; Mazurek et al., 2014), which could suggest that people with lower SES have less access to services and are diagnosed later. However, Brett et al. (2016) reported that those with autism who were younger than 60 months had overall lower SES than those over 60 months. Our results suggest that those who suspected a diagnosis did not differ in SES from those who received a diagnosis, making it unlikely that SES was a reason that no diagnosis was received.

**Discussion**

**Adults in household**

Across OEEF-Lab/Uni, SDDS-Online/Uni and Prolific-Online/Crowd-FU, 18 people reported there was only one adult in their household (11 no-FH, 7 FH-any), versus 624 families with 2 or more adults (587 no-FH, 37 FH-any). The chi-square test was significant (χ^2^ = 29.77, *p* < .001; OR 10.10).

References

Brett, D., Warnell, F., McConachie, H., & Parr, J. R. (2016). Factors affecting age at ASD diagnosis in UK: No evidence that diagnosis age has decreased between 2004 and 2014. *Journal of Autism and Developmental Disorders*, *46*(6), 1974-1984. <https://doi.org/10.1007/s10803-016-2716-6>

Hosozawa, M., Sacker, A., Mandy, W., Midouhas, E., Flouri, E., & Cable, N. (2020). Determinants of an autism spectrum disorder diagnosis in childhood and adolescence: Evidence from the UK Millennium Cohort Study. *Autism*, *24*(6), 1557-1565. <https://doi.org/10.1177/1362361320913671>

Mazurek, M. O., Handen, B. L., Wodka, E. L., Nowinski, L., Butter, E., & Engelhardt, C. R. (2014). Age at first autism spectrum disorder diagnosis: the role of birth cohort, demographic factors, and clinical features. *Journal of Developmental & Behavioral Pediatrics*, *35*(9), 561-569. <https://doi.org/10.1097/DBP.0000000000000097>

Miller, M., Musser, E. D., Young, G. S., Olson, B., Steiner, R. D., & Nigg, J. T. (2019). Sibling recurrence risk and cross-aggregation of attention-deficit/hyperactivity disorder and autism spectrum disorder. *JAMA pediatrics*, *173*(2), 147-152. <https://doi.org/10.1001/jamapediatrics.2018.4076>
